# Healthy goats naturally devoid of prion protein

**DOI:** 10.1186/1297-9716-43-87

**Published:** 2012-12-18

**Authors:** Sylvie L Benestad, Lars Austbø, Michael A Tranulis, Arild Espenes, Ingrid Olsaker

**Affiliations:** 1 Norwegian Veterinary Institute, P.O.Box 750, Sentrum, Oslo, 0106, Norway; 2 The Norwegian School of Veterinary Science, P.O.Box 8146 Dep, Oslo, 0033, Norway

## Abstract

Prion diseases such as scrapie in small ruminants, bovine spongiform encephalopathy (BSE) in cattle and Creutzfeldt-Jakob disease (CJD) in man, are fatal neurodegenerative disorders. These diseases result from the accumulation of misfolded conformers of the host-encoded prion protein (PrP) in the central nervous system. To date naturally-occurring PrP free animals have not been reported. Here we describe healthy non-transgenic animals, Norwegian Dairy Goats, lacking prion protein due to a nonsense mutation early in the gene. These animals are predicted to be resistant to prion disease and will be valuable for research and for production of prion-free products.

## Introduction, methods and results

Transmissible spongiform encephalopathies (TSE) or prion diseases such as Creutzfeldt-Jakob disease (CJD) in man, bovine spongiform encephalopathy (BSE) in cattle, and scrapie in sheep and goats are invariably fatal diseases. These diseases may be inherited (mutations in the prion protein gene), sporadic (unknown cause) or acquired (transmission of infectious agent). They are characterized by the accumulation of conformational isoforms of the normal cellular prion protein (PrP) in the central nervous system [1]. According to the “protein-only” hypothesis, the partly protease-resistant, misfolded PrP, designated PrP^Sc^, is the disease agent [2]. Prion diseases are entirely dependent on endogenous PrP expression. For instance, PrP gene knockout mice are resistant to prion disease [3], and further studies of transgenic mice have shown that the level of PrP expression is inversely correlated with the incubation time [4,5]. In addition, knockdown of the PrP expression by RNAi rescues early neuronal dysfunction and prolongs survival time in mice with prion disease [6].

As with sheep, goats are susceptible to both classical [7-11] and atypical [10,12-14] scrapie. One goat has also been identified as infected with BSE [15]. Classical scrapie in goat has been reported in the USA, Canada and many European countries, and its prevalence is particularly high in Cyprus (accounting for 85% of all cases in the EU between 2002 and 2007) [10]. As described in many other species, the goat PrP gene (*PRNP*) is polymorphic and some variants have been associated with resistance to the disease [10,11,16-18].

The present study is based on the discovery, in the indigenous goat breed Norwegian Dairy Goat originating from the north-European Landrace, of a new variant of the *PRNP* displaying a premature stop codon. Additional goats were subsequently screened for *PRNP* polymorphisms and one goat homozygous for the new variant was analysed for the presence of PrP. Animal experiments described in this paper have been subjected to stringent ethical evaluation and were carried out in compliance with institutional and national regulations.

Genomic DNA was extracted from brain or blood samples using DNeasy 96 blood and tissue kit (Qiagen) according to the suppliers’ protocol. A fragment covering the coding region of the goat *PRNP* was amplified by PCR with M13 tailed primers GoatF1: M13-21-CAGTCATTCATTATGCTGCAGACTT (this work) and BilG2-R: M13rev-CTATCCTACTATGAGAAAAATGAG [19] and analysed by sequencing with Big Dye Primer chemistry (Applied Biosystems, Foster City, USA).

**Figure 1:**
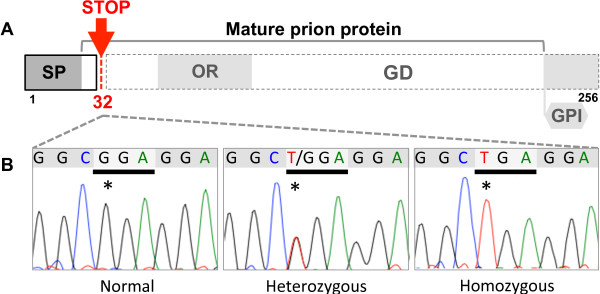
**Graphical overview of the prion protein structure.** Panel **A**: Goat PrP with structural domains: signal peptide (SP), five octapeptide repeats (OR), globular domain (GD) and glycophosphatidylinositol-anchor (GPI). Panel **B**: Sequences of the goat *PRNP*-region with the polymorphic base (stars).

The genetic survey of the Norwegian Dairy Goat population revealed the presence of the naturally occurring nonsense mutation in the *PRNP* at a surprisingly high allele frequency (11%, *n*=192, comprising samples of unrelated animals from several flocks and random brain samples collected as part of the Norwegian scrapie surveillance programme). The mutation was located in codon 32 (_32_Stop) terminating the PrP synthesis, leaving only seven amino acids of the mature protein (Figure 1). A subsequent search among the offspring of heterozygous animals identified two homozygous _32_Stop goats (twins born in March 2009). One goat was sacrificed at 30 months of age and the other is currently 3.5 years old (October 2012) and remains alive in a research herd. The *PRNP* sequence of the sacrificed goat has been submitted to GenBank (Acc. no KC145281). Both goats have given birth to healthy kids (twice and three times, respectively) and none of them have shown any abnormal behaviour or other characteristics distinct from their flock-mates. The sacrificed goat was subjected to a routine clinical and neurological examination, then euthanized and necropsied. No abnormalities were noted.

**Figure 2:**
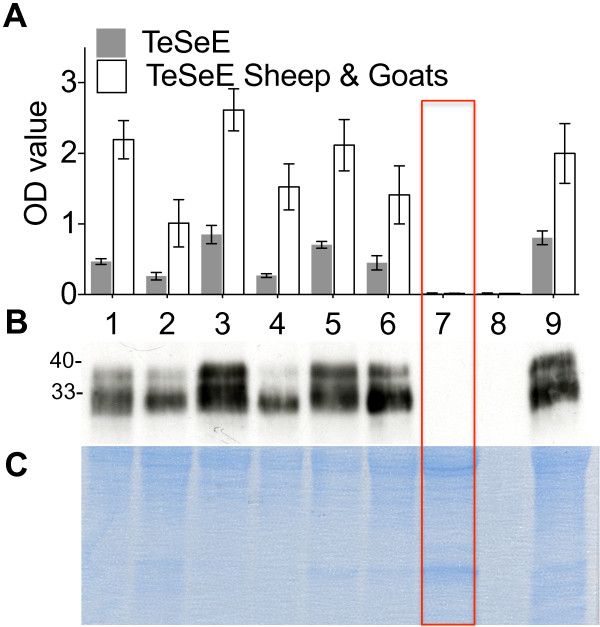
**Detection of PrP in goat brain homogenates.** Sample 7, red box: the homozygous _32_Stop goat, Sample 1–6 and 9: wild type goats including two heterozygous _32_Stop goats (2 and 6), Sample 8: negative control, normal goat brain digested with proteinase K. MW: Molecular weight in kilodaltons. **A**) Modified ELISA (TeSeE and TeSeE Sheep & Goats, Bio-Rad). Each Optical Density (OD) value represents the mean ± SEM of five different measures per sample. The homozygous _32_Stop goat displayed OD values in the same range as the negative control (< 0.03), and far under the normal cut off value (approximately 0.230 and 0.150 for TeSeE and TeSeE Sheep & Goats respectively). **B**) Western blot (TeSeE WESTERN BLOT, Bio-Rad). Note that PrP was not identified in the homozygous _32_Stop goat and the negative control sample. **C**) Protein stain of the gel in panel B. Normal proteins are present in all samples, with the exception of sample 8, the negative control where proteins were digested by proteinase K.

To determine the presence or absence of PrP, goat brains were analysed by Western blotting (WB) in two separate laboratories with different protocols and by Enzyme-Linked Immunosorbent Assay (ELISA). In laboratory 1, slightly modified ELISA (TeSeE and TeSeE Sheep and Goat, Bio-Rad, Marnes-La-Coquette, France) and WB tests (TeSeE Bio-Rad) were used on the brain homogenates as recommended by the producers. Since the goal was to evaluate the presence of normal PrP and not only protease-resistant PrP^Sc^, proteinase K (PK) hydrolysis of the samples was omitted, except in the negative control (sample 8 in Figure 2). In laboratory 2, the following WB method was used. Brain samples were homogenized at 4°C by a Dounce all-glass homogenizer in lysis buffer (LB) containing Tris–HCl pH 7.4, 150 mM NaCl, 0.5% (w/v) Triton X-100, 0.5% sodium deoxycholate, 1mM EDTA and proteinase inhibitor tablets (Roche, Applied Science, Indianapolis, USA). Samples were centrifuged at 1000 × g for 10 min to remove debris. Prior to SDS electrophoresis samples were boiled for 5 min in 4 × SDS-samples buffer (Invitrogen, Oslo, Norway), supplemented with a reducing agent and centrifuged at 13 000 × g for 5 min. Samples were run on 12% pre-cast polyacrylamide gels (Bio-Rad) with Tris MOPS as running buffer and transferred to polyvinylidene fluoride membranes by a semi-dry blotter. Membranes were blocked for 1 h in 5% fat-free dry milk dissolved in Tris buffered saline (TBS), prior to incubation overnight at 4°C with primary antibodies SAF32 (SPI-Bio, Pas-Du-Lac, France) or P4 (R-Biopharm, Darmstadt, Germany) at a concentration of 0.04 μg/mL. Secondary antibody (goat anti-mouse) labelled with alkaline phosphatase (GE Healthcare, Little Chalfont, Buckinghamshire, UK) was used to visualize bands with a fluorescence imager (Typhoon 9200, GE Healthcare) after incubation with the ALP substrate (ECFTM, Western blotting reagent pack, GE Healthcare). As internal controls, total proteins were stained in gel with Gel CodeTM Blue Safe Protein Stain (Thermo Scientific, Oslo, Norway) or with Biosafe Coomassie (Bio-Rad) in laboratory 1 and 2 respectively, as described by the manufacturers.

**Figure 3:**
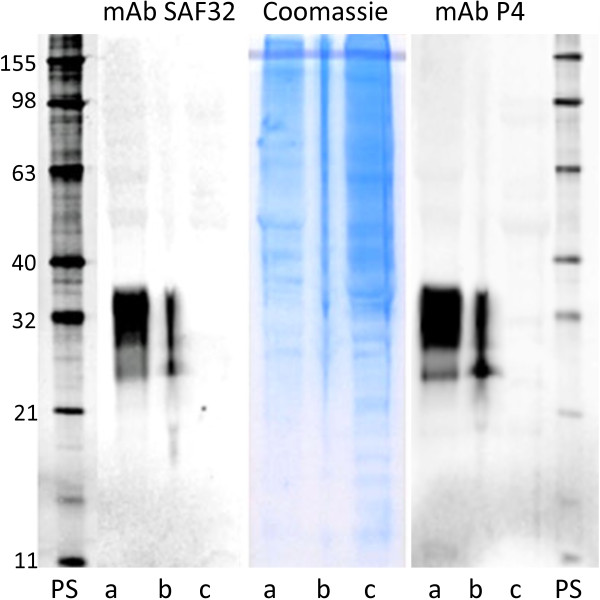
**Detection of PrP by Western blot.** Brain samples from goats analysed using the monoclonal antibodies SAF32 (left panel) and P4 (right panel). PrP is detectable in the two wild type goats (lane a and b) but not in the homozygous _32_Stop goat, (lane c). The central panel shows the presence of normal brain proteins in the three samples (Coomassie stain). PS: protein standards (kilodaltons).

As an expected consequence of the early stop codon in the gene, PrP could not be detected in the homozygous _32_Stop animal by any of the methods, whereas it was clearly demonstrated in all the control goats (Figures 2 and 3).

## Discussion

Whilst experimental production of PrP gene knockouts of cattle [20] and goats [21,22] has been described, this is the first description of apparently healthy non-transgenic animals lacking PrP due to a naturally-occurring mutation. Whether the _32_Stop haplotype is unique to Norwegian Dairy Goats is not known. Several PrP polymorphisms have been described in goats [10,11,17], but only one animal heterozygous for a nonsense mutation late in the goat *PRNP,* at codon 163, has been reported [11].

The _32_Stop goats are unique natural tools for investigating how the PrP may be involved in both physiological and pathological processes. PrP is a cell surface protein largely expressed within the nervous system and is highly conserved across mammalian species. However, the natural function of the protein remains elusive. Similar to transgenic PrP gene knockout animals [1,20,21], the identified goats homozygous for the _32_Stop haplotype lack PrP, yet are viable and appear normal. This suggests that the protein is not involved in vital physiological processes or that its absence can be compensated for by other mechanisms. In disease-related processes PrP might be both positively and negatively involved. Studies of transgenic animals suggest that PrP has a role in tissue repair and protection against cellular stress [1,23]. Recent reports also indicate that PrP is involved in mediating Aβ-induced cytotoxicity in Alzheimer’s disease [24-26].

In conclusion, this is the first description of naturally-occurring mammals lacking PrP. Goats lacking PrP differ from the valuable PrP knockout mice in many aspects and might provide a supplementary model for investigating the function of the protein. Additionally, goats resistant to prion disease will be valuable not only for breeding but also for production of prion-free materials.

## Competing interests

The authors declare that they have no competing interests.

## Authors’ contributions

All the authors contributed to the general interpretation of the data and the writing of the manuscript. In addition, SLB designed the experiments and supervised the ELISA and Western blot tests. LA designed the experiments, interpreted the data, performed and interpret the sequencing and genetic analyses and designed the figures. MAT supervised the Western blot tests. AE participated in the collection of the material. IO discovered the stop mutation, designed the experiments, supervised and interpreted the genetic analyses. All authors read and approved the final manuscript.
